# Scar tenderness as a sensitive marker for uterine scar dehiscence or uterine rupture

**DOI:** 10.12669/pjms.42.8.7502

**Published:** 2026-08

**Authors:** Maria, Erum Majid, Tanzila Fahim, Bader Faiyaz Zuberi, Saima Mujtaba

**Affiliations:** 1 Maria, Ward 9B, Department of Gynaecology & Obstetrics, Jinnah Postgraduate Medical Centre, Karachi, Pakistan; 2 Erum Majid, Ward 9B, Department of Gynaecology & Obstetrics, Jinnah Postgraduate Medical Centre, Karachi, Pakistan; 3 Tanzila Fahim, Ward 9B, Department of Gynaecology & Obstetrics, Jinnah Postgraduate Medical Centre, Karachi, Pakistan; 4 Bader Faiyaz Zuberi, OMI Hospital, Depot Lines, M A Jinnah Road, Karachi, Pakistan; 5 Saima Mujtaba, Ward 9B, Department of Gynaecology & Obstetrics, Jinnah Postgraduate Medical Centre, Karachi, Pakistan

**Keywords:** Caesarean Section, Scar Tenderness, Scar dehiscence, Uterine Rupture

## Abstract

**Objective:**

To determine the sensitivity, specificity, and diagnostic accuracy of caesarean scar tenderness in predicting uterine dehiscence or rupture during per-operative findings.

**Methodology:**

This cross-sectional study was conducted in the Department of Obstetrics & Gynaecology, JPMC, Karachi, from November 2021 to July 2024. Women with singleton pregnancies having previous caesarean were given Trial of Labor After Caesarean (TOLAC) after informed consent using consecutive sampling. Exclusion criteria included uterine abnormalities, hypertension, diabetes mellitus, multiple pregnancies, and pre-eclampsia. Data were collected on demographic details, clinical findings, and operative outcomes. Statistical analysis was performed using SPSS Version 27.

**Results:**

A total of 385 women were included in the study. Scar tenderness was present in 67.8% of women. Uterine rupture was found in 22.3% of cases, while scar dehiscence was present in 49.9%. The sensitivity of scar tenderness in predicting uterine rupture and dehiscence was 85.6%, and the specificity was 78.5%. The positive predictive value (PPV) was 91.2%, and the negative predictive value (NPV) was 67.7%. The overall diagnostic accuracy was 83.6%.

**Conclusions:**

Scar tenderness is a useful clinical predictor of uterine scar complications during TOLAC, particularly in low-resource settings.

## INTRODUCTION

Globally, trend of caesarean section (CS) has been rising steadily, and this trend is predicted to continue in the coming years. Asian countries, particularly low and middle-income countries, may face increasing morbidity and mortality related to overuse of caesarean section.^1^ Rising caesarean section rates may also compromise quality of care, strain resources, and contribute to preventable adverse maternal outcomes.

Global effective interventions are need of the time to reverse the trend. Addressing the CS problem thoroughly is a worldwide priority if the Sustainable Development Goals for safe surgery and anaesthesia are to be met by 2030.^2^ As the prevalence of CS continues to rise, the incidence of scar-related complications, including scar dehiscence, is becoming increasingly common.^3^ Scar dehiscence is a significant complication of CS, occurring in up to 6.2 per 1000 trial of labor.^4^ A study conducted at JPMC revealed that the majority of CS fall under Robson Classification-5, highlighting the growing concern of complications associated with CS deliveries.^5^ While vaginal birth after CS (VBAC) has a success rate of 70%, it necessitates rigorous monitoring, particularly in emergency situations.^6^

Thinning of the uterine scar can lead to compromised integrity of the uterine walls, making them more susceptible to infection and scar dehiscence. This can result in severe consequences, including increased risk of maternal and foetal morbidity, as well as the need for a second laparotomy.^7^,^8^

Despite advances in imaging technology, the diagnosis of scar dehiscence remains a clinical challenge. The development of a reliable and effective method for predicting scar integrity is essential for improving patient outcomes and reducing healthcare costs. Several studies have demonstrated that monitoring scar tenderness and maternal pulse is a reliable and effective method for predicting scar integrity.^9^,^10^ In fact, a recent study concluded that monitoring scar tenderness and pulse is an important tool for assessing scar integrity in patients with a history of CS.^11^

Scar dehiscence in patients of previous caesarean section is a serious complication because if not predicted it can lead to uterine rupture with serious maternal and perinatal morbidity and mortality.^12^ Risk factors for uterine rupture include previous CS, grand multiparity, instrumental delivery, and use of uterotonic drugs to induce or augment labour.^13^

The goal of this study was to provide evidence for the diagnostic accuracy of scar tenderness as a rapid, non-invasive, and cost-effective method to detect uterine rupture, which could be integrated into routine clinical practice to improve outcomes. The findings of this study have the potential to improve clinical practice and patient care.

This study will determine the sensitivity and specificity and diagnostic accuracy of caesarean scar tenderness in predicting uterine dehiscence or rupture on per-operative findings.

### Operational Definitions:

### Scar Tenderness:

will be considered positive if wink response of patient was observed after pressing below pubic symphysis in between uterine contractions while engaging the woman in conversation and noting for a visible wince. This will be checked by two trained consultants independently to reduce inter observer variability.

### Dehiscence:

Uterine dehiscence was defined by incomplete division of the uterine wall that does not encompass all uterine layers.

### Uterine Rupture:

Uterine rupture was defined as complete disruption of all layers of the uterus- the endometrium, myometrium and perimetrium.

### Diagnostic Accuracy:

was assessed as per sensitivity, specificity, positive and negative predictive value as follows:

**Table:** 

Independent Variable	Dependent Variable	
	Positive	Negative
Positive	A	B
Negative	C	D

Cell a: True Positive (TP), Cell b: False Positive (FP),

Cell c: False Negative (FN), Cell d: True Negative (TN).


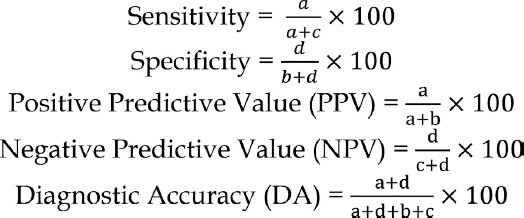


## METHODOLOGY

This cross-sectional study was conducted in Department of Obstetrics & Gynaecology, JPMC, Karachi during the period November 2021 and July 2024. All women with singleton pregnancy having previous caesarean scar were included by consecutive sampling after informed consent. Other inclusion criteria included cephalic presentation, maternal age 25-45 years and spontaneous labour at term. Patients with uterine abnormalities, hypertension, diabetes mellitus, multiple pregnancies and pre-eclampsia were excluded as they can cause complications from previous surgery and are potential confounders. Sample size was calculated to obtain a two-sided 95% confidence interval for a single proportion. The Exact (Clopper-Pearson) formula was used to calculate the confidence interval. The sample proportion is assumed to be 0.2 as reported by Vandenberghe G et al for Asean population.^14^ To produce a confidence interval with a width of no more than 0.2, 370 subjects will be needed. The sample size was computed using PASS 2026, version 26.0.3.

### Ethical Approval:

Ethical approval was taken from IRB of JPMC (NP.F.2-81/2021/57185/JPMC) dated March 31, 2021.

### Data collection:

The demographic details of each patient were recorded, including clinical findings, Trial of Labor After Caesarean (TOLAC) was monitored by principal investigator. Monitoring of trial of scar was done by observation of scar tenderness, maternal pulse and blood pressure. Assessment for cervical changes was done every four hours or as required. TOLAC was terminated for scar tenderness, unexplained maternal tachycardia, fresh vaginal bleeding, foetal heart rate less than 110 and non-progress of labour. Operative findings such as uterine dehiscence and uterine rupture and outcome were also evaluated as per operational definition. All data such as age, birth weight, GA, education, socioeconomic status, booking status, comorbid & parity was recorded on a predesigned proforma. Confounding variables and biasness were controlled by strictly following the inclusion criteria.

### Data analysis:

Data were analysed using IBM SPSS Statistics for Windows, Version 27.0 (IBM Corp., Armonk, NY, USA). Frequency and percentage were computed for qualitative variables like clinical findings (such as maternal pulse, scar tenderness), uterine rupture & dehiscence. For calculation of sensitivity/specificity, both uterine rupture & dehiscence, were clubbed together. Mean ±SD was calculated for quantitative variable, i.e., age, birth weight, gestational age, while frequencies were calculated for parity, previous caesarean delivery, scar tenderness, uterine rupture & dehiscence. A 2 x 2 table was generated to calculate the sensitivity, specificity, positive predictive value, negative predictive values, and the diagnostic accuracy of scar tenderness by formulas mentioned in operational definitions. Two separate models were run for Multiple Regression analysis taking Uterine Rupture and Scar Dehiscence as dependent variables while birth weight, gestational age, previous caesareans, parity and maternal age were taken as independent variables. R^2^ and adjusted R^2^ values were reported. Significance was set at ≤.05.

## RESULTS

A total of 385 women satisfying inclusion/exclusion criteria and willing to consent were included in the study. Mean age of included women was 27.9 ±2.3 years. Mean gestational age was 39.0 ±0.61 weeks, parity 2.16 ±0.97, birth weight 3.04 ±0.15 kg. Scar tenderness was present in 261 (67.8%) of women. On surgery uterine rupture was found in 86 (22.3%) while scar dehiscence was present in 92 (23.9%) while normal uterus was found in 207 (53.8%) of cases. Details are given in Table-I.

**Table-I:** Demographic details.

Variable		Mean	±SD
Age (years)		27.85	2.26
Gestational Age (weeks)		39.00	.61
Birth Weight (kg)		3.04	.15
*Variable*	*Category*	*Count*	*%*
Parity	1	117	30.39%
	2	129	33.51%
	3	101	26.23%
	4	38	9.87%
Previous Caesarean	1	216	56.10%
	2	119	30.91%
	3	50	12.99%
Scar Tenderness	Present	261	67.79%
	Absent	124	32.21%
Diagnosis	Normal Uterus	207	53.76%
	Uterine Rupture	86	22.34%
	Scar Dehiscence	92	23/89%
Combined Uterine Rupture & Scar Dehiscence	Present	278	72.21%
	Absent	107	27.79%

Scar tenderness was present in 90.7% in uterine rupture, 65.2% in scar dehiscence and in 59.4% of normal uterus. Significant differences in frequencies were found by χ^2^-test, details are given in Table-II. Mean maternal age, birth weight and gestational age were compared by presence or absence of scar tenderness by Students’ t-test. No significant differences were found, details in Table-III.

**Table-II:** Cross tabulation of Scar Tenderness, Previous C-Section & Parity with Uterine Rupture, Dehiscence & Normal uterus by χ^2^ test.

		Uterine Rupture		Scar Dehiscence		Normal Uterus		Sig.
		N	%	N	%	N	%	
Scar Tenderness	Present	78	90.7%	160	83.3%	23	21.5%	<.001
	Absent	8	9.3%	32	16.7%	84	78.5%	
Previous C-Section	1	38	44.2%	88	45.8%	90	84.1%	<.001
	2	35	40.7%	71	37.0%	13	12.1%	
	3	13	15.1%	33	17.2%	4	3.7%	
Parity	1	3	3.5%	63	32.8%	51	47.7%	<.001
	2	9	10.5%	94	49.0%	26	24.3%	
	3	50	58.1%	35	18.2%	16	15.0%	
	4	24	27.9%	0	0.0%	14	13.1%	

Significance ≤.05.

**Table-III:** Comparison of means of age, birth weight & gestational age with scar tenderness by Students’ t-test.

	Scar Tenderness	N	Mean	±SD	Sig
Age (years)	Present	261	27.98	2.214	.105
	Absent	124	27.58	2.345	
Birth Weight (kg)	Present	261	3.045	.1458	.451
	Absent	124	3.033	.1513	
Gestational Age (weeks)	Present	261	38.985	.6200	.551
	Absent	124	39.024	.5768	

Significance ≤.05.

Maternal age, birth weight and gestational age were compared by per-operative uterine status, and maternal age was found significantly more in women with scar dehiscence while no significant difference was found in birth weight and gestational age (Table-IV). Calculation of sensitivity and specificity was done by creating 2x2 table, true positive were 238 (61.8%), true negative was 84 (21.8%), false positive were 23 (6.0%) and false negative were 40 (10.4%). Sensitivity was calculated as 85.6% while specificity was 78.5%. PPV of scar tenderness was 91.2% and NPV was 67.7% (Table-V). Diagnostic accuracy of scar tenderness was 83.6%.

**Table-IV:** Comparison of Age, Birth Weight & Gestational Age with Uterine Rupture, Dehiscence & Normal uterus by ANOVA.

	Uterine Rupture		Scar Dehiscence		Normal Uterus		Sig.
	Mean	±SD	Mean	±SD	Mean	±SD	
Age (years)	27.30	2.11	28.04	2.15	27.96	2.51	.036
Birth Weight (kg)	3.04	.16	3.05	.14	3.02	.15	.174
Gestational Age (weeks)	39.07	.55	38.96	.64	39.00	.58	.402

Significance ≤.05.

**Table-V:** Cross tabulation of scar tenderness with combined uterine outcome by χ^2^ test and Sensitive & Specificity Testing.

		Scar Tenderness		Total
		Present	Absent	
Combined UR & SD	Present	238 TP	23 FP	261
	Absent	40 FN	84 TN	124
Total		278	107	385

TP = True Positive; FP = False Positive;

FN = False Negative; TN = True Negative.

### Predictors of Uterine Rupture:

A multiple linear regression was conducted to assess whether age, scar tenderness, birth weight, gestational age, previous cesarean section, and parity predicted uterine rupture. The model was statistically significant, F (6, 378) = 40.79, *p* < .001, explaining 39.3% of the variance (R² = .393, adjusted R² = .383).

Significant predictors included scar tenderness (B = 0.27, SE = 0.04, β = .31, ***p*** < .001), previous cesarean section (B = 0.14, SE = 0.03, β = .24, ***p*** < .001), parity (B = –0.26, SE = 0.02, β = –.61, ***p*** < .001), and age (B = 0.02, SE = 0.01, β = .09, ***p*** = .027). Birth weight (p = .107) and gestational age (p = .059) were not significant predictors. Details are given in Table-VI.

**Table-VI:** Multi-Variate Regression Table for Dependent Variable Uterine Rupture.

Predictor	B	SE	β	t	p
Constant	2.733	1.148	—	2.380	.018
Age (years)	0.017	0.008	.090	2.214	.027
Scar tenderness	0.273	0.039	.306	7.071	<.001
Birth weight (kg)	0.198	0.123	.070	1.616	.107
Gestational age (weeks)	–0.052	0.028	–.076	–1.894	.059
Previous CS	0.142	0.029	.242	4.841	<.001
Parity	–0.262	0.019	–.609	–13.531	<.001

***Model summary:*** R = .627, R² = .393, Adjusted R² = .383, F(6, 378) = 40.79, p < .001.

### Predictors of Scar Dehiscence:

A second multiple regression examined the same predictors for scar dehiscence. The model was significant, **F**(6, 378) = 6.56, ***p*** < .001, but explained only 9.4% of the variance (R² = .094, adjusted R² = .080).

Significant predictors included birth weight (B = –0.39, SE = 0.15, β = –.14, p = .011) and parity (B = 0.13, SE = 0.02, β = .30, p < .001). Age, scar tenderness, gestational age, and previous cesarean section were not significant predictors. Table-VII.

**Table-VII:** Multi-Variate Regression Table for Dependent Variable Scar Dehiscence.

Predictor	B	SE	β	t	p
Constant	3.938	1.436	—	2.742	.006
Age (years)	–0.010	0.009	–.053	–1.061	.289
Scar tenderness	–0.042	0.048	–.046	–0.871	.384
Birth weight (kg)	–0.392	0.154	–.135	–2.552	.011
Gestational age (weeks)	–0.022	0.035	–.031	–0.630	.529
Previous CS	–0.057	0.037	–.095	–1.556	.121
Parity	0.133	0.024	.302	5.496	<.001

***Model summary:*** R = .307, R² = .094, Adjusted R² = .080, F(6, 378) = 6.56, p < .001.

## DISCUSSION

The present study demonstrated that scar tenderness had substantial diagnostic utility for detecting combined uterine rupture and scar dehiscence among women undergoing TOLAC.^13^ The high positive predictive value suggests that the presence of scar tenderness was strongly associated with abnormal intra-operative scar findings in this cohort. Conversely, the comparatively modest negative predictive value indicates that absence of tenderness should not be interpreted as sufficient evidence to exclude uterine scar pathology, particularly in high-risk obstetric settings. This interpretation is consistent with contemporary VBAC guidance, which emphasizes continuous clinical surveillance, facility readiness for emergency caesarean delivery, and integration of maternal and foetal warning signs during TOLAC.^9^,^10^,^13^,^15^,^16^

The sensitivity observed in this study is comparable with the findings of Khalil et al., who reported a sensitivity of 86.3% and specificity of 86.0% for clinical scar tenderness in predicting scar strength among women undergoing lower-segment caesarean section.^17^ This concordance supports the reproducibility of scar tenderness as an important clinical warning sign. However, the negative predictive value reported by Khalil et al.^17^ was considerably higher than that observed in the present study. This discrepancy may be attributable to differences in case mix, baseline prevalence of scar complications, operative thresholds, and the present study’s use of a combined outcome comprising both uterine rupture and scar dehiscence. In contrast, Gaikwad et al. reported higher sensitivity but markedly lower specificity and diagnostic accuracy, indicating that scar tenderness may function primarily as a sensitive screening sign, while its specificity is influenced by clinical context, patient selection, and the diagnostic criteria used to define scar complications.^10^

The interpretation of scar tenderness should, nevertheless, remain integrated within the broader clinical assessment. Liaqat and Qazi observed that scar tenderness alone was not significantly associated with scar complications, whereas the coexistence of scar tenderness, unexplained maternal tachycardia, and abnormal cardiotocography was strongly associated with scar-related morbidity during TOLAC.^9^ This is contrary to the present findings, in which scar tenderness remained an independent predictor of uterine rupture in regression analysis. The difference may reflect the larger sample size of the current study, intra-operative confirmation of outcomes, and the higher burden of scar abnormalities in a tertiary-care referral population. Taken together, these findings suggest that scar tenderness should prompt immediate reassessment but should not be used in isolation; rather, it should be interpreted alongside maternal pulse, foetal heart rate pattern, labour progress, vaginal bleeding, and overall clinical status.

Compared with studies evaluating sonographic lower uterine segment assessment, the diagnostic performance of scar tenderness in the present study was relatively lower. Cui and Wu reported sensitivity of 100.0%, specificity of 91.8%, diagnostic accuracy of 92.5%, positive predictive value of 55.6%, and negative predictive value of 100.0% for ultrasound assessment of lower uterine segment dehiscence and rupture among women with a previous caesarean section.^18^ These findings are supported by a meta-analysis by Kok et al., which concluded that antenatal lower uterine segment measurement is a promising method for predicting uterine defects during trial of labour after caesarean section, although heterogeneity in measurement technique and cut-off values limits universal application.^15^ More recent prospective and secondary analyses have also suggested that lower uterine segment thickness stratification may contribute to risk assessment during TOLAC, particularly when combined transabdominal and transvaginal measurements are used.^19^,^20^ However, ultrasonography is resource-dependent and may not be feasible in all intrapartum settings. Scar tenderness, by contrast, is immediate, inexpensive, repeatable, and requires no equipment. Therefore, it may be best conceptualized as a pragmatic clinical screening tool rather than a substitute for sonographic evaluation, cardiotocography, or comprehensive obstetric assessment.

The frequency of uterine rupture and scar dehiscence observed in the present study was higher than that reported in several population-based TOLAC cohorts. In this study, uterine rupture was identified in 22.3% of cases and scar dehiscence in 23.9% of cases. By comparison, population-based data from Stockholm-Gotland, Roeck Hansen C et al reported uterine rupture in 1.8% of women undergoing TOLAC after one previous caesarean section, with induction of labour and prostaglandin use identified as important contributors to risk.^13^ Similar population-based and tertiary-centre cohorts have reported substantially lower rupture rates during TOLAC, generally below 1% to 2%, particularly when women are carefully selected and monitored in appropriately equipped facilities.^13^,^16^ Similarly, Savukyne et al. described symptomatic uterine rupture as an uncommon but clinically serious complication in a fifteen-year review.^21^ The higher frequency in the current cohort is likely explained by referral bias, tertiary-care case selection, inclusion of women requiring operative confirmation following TOLAC monitoring, and the combined evaluation of rupture and dehiscence rather than estimation of population-level incidence.

Multivariable analysis in the present study identified scar tenderness, previous caesarean section, parity, and maternal age as significant predictors of uterine rupture. These findings are broadly consistent with Overtoom et al., who reported previous caesarean section, higher maternal age, gestational age below 37 weeks, labour augmentation, and migration background from Sub-Saharan Africa or Asia as risk indicators for adverse outcomes associated with uterine rupture.^22^ The association between prior caesarean delivery and uterine rupture is also supported by recent population-based data, which describe previous caesarean section as the primary risk factor for rupture during TOLAC and demonstrate increased risk with induction, prostaglandins, and augmentation of labour.^13^,^23^ The association between maternal age and uterine rupture in the current study is consistent with this prior evidence. Gestational age, however, was not a significant predictor in our model, which may be due to the relatively narrow gestational-age distribution of the study population, as most women presented at term.

Birth weight was not a significant predictor of uterine rupture in the present model, although it was significantly associated with scar dehiscence. This finding differs from Arusi et al., who reported foetal weight greater than 3.8 kg, inadequate antenatal care, labour duration exceeding 15 hours, and previous successful vaginal delivery as factors associated with uterine rupture after trial of labour.^24^ The absence of a significant association with rupture in the present study may reflect the relatively low and narrow birth-weight distribution, with a mean birth weight of 3.04 kg. Conversely, the association between birth weight and scar dehiscence is supported by Tyagi et al., who identified foetal weight greater than 3 kg as a significant predictor of scar dehiscence.^25^ Additional studies of scar dehiscence have reported associations with short inter-pregnancy or inter-delivery interval, emergency caesarean delivery, multiple previous caesarean sections, and preterm delivery, indicating that scar dehiscence is likely multifactorial rather than determined by foetal size alone.^26^,^27^ This may indicate that foetal size contributes more consistently to progressive scar thinning or partial separation than to complete rupture, although this interpretation requires confirmation in prospective multicenter studies.

### Limitations:

It was conducted at a single tertiary-care centre using consecutive sampling; therefore, the findings may not be generalizable to all obstetric populations, especially primary-care or private-sector settings. Scar tenderness is a clinical sign and, despite assessment by trained consultants, some subjectivity and inter-observer variation may remain. In addition, important intrapartum factors such as duration of labour, augmentation, cervical progress, and detailed foetal monitoring patterns were not fully evaluated in the regression models.

## CONCLUSIONS

Scar tenderness showed good diagnostic performance for detecting combined uterine rupture and scar dehiscence among women undergoing TOLAC, with sensitivity of 85.6%, specificity of 78.5%, positive predictive value of 91.2%, negative predictive value of 67.7%, and overall diagnostic accuracy of 83.6%. Scar tenderness, previous caesarean section, parity, and maternal age were significant predictors of uterine rupture, whereas parity and birth weight were significant predictors of scar dehiscence.

### Authors’ Contribution:

**M:** Literature search, Did data collection. **EM:** Idea conception. Critical Review, **TF:** Literature search, Initial manuscript writeup.

**BFZ:** Statistical analysis and manuscript review and editing. **SM:** Critical Review**,** Final approval of manuscript.

All authors are responsible for accuracy and integrity of work.
